# Scintillation Properties of Lanthanide Doped Pb_4_Lu_3_F_17_ Nanoparticles

**DOI:** 10.3390/ma16031147

**Published:** 2023-01-29

**Authors:** Peng Qiao, Yiheng Ping, Hongping Ma, Lei Lei

**Affiliations:** 1 Zhejiang Academy of Special Equipment Science, Hangzhou 310018, China; 2 Key Laboratory of Special Equipment Safety Testing Technology of Zhejiang Province, Hangzhou 310018, China; 3 School of Mechanical and Energy Engineering, Zhejiang University of Science and Technology, Hangzhou 310023, China; 4 College of Optical and Electronic Technology, China Jiliang University, Hangzhou 310018, China

**Keywords:** Pb_4_Lu_3_F_17_, X-ray, rare earth, CsI (TI), energy transfer

## Abstract

Inorganic scintillators are of great significance in the fields of medical CT, high-energy physics and industrial nondestructive testing. In this work, we confirm that the Pb_4_Lu_3_F_17_: Re (Re = Tb, Eu, Sm, Dy, Ho) crystals are promising candidates for a new kind of scintillator. Detailed crystal structure information is obtained by the Rietveld refinement analysis. Upon X-ray irradiation, all these scintillators exhibited characteristic 4f-4f transitions. The Ce and Gd ions were verified to be useful for enhancing the scintillation intensity via introducing energy transfer processes. The integrated scintillation intensity of the Pb_4_Lu_3_F_17_: Tb/Ce is about 16.8% of the commercial CsI (Tl) single crystal. Our results manifested that Pb_4_Lu_3_F_17_: Re has potential application in X-ray detection and imaging.

## 1. Introduction

Scintillator is a kind of luminescent material that can effectively absorb ionizing radiation and convert the absorbed radiation energy into visible light [1,2,3,4,5]. Inorganic scintillating materials have been broadly used in many fields, such as medical CT [6,7], high-energy physics [8,9] and industrial nondestructive testing [10,11]. Conventional oxide scintillators, such as Bi_4_Ge_3_O_12_ (BGO) [12], PbWO_4_ (PWO) [13] and Lu_2_SiO_5_: Ce (LSO: Ce) [14] show fast response and good chemical stability, while the halide scintillators, such as NaI (TI), CsI (TI) [15], LaCl_3_: Ce [16] and CaF_2_: Ce [17], have high luminous efficiency. For example, BaY_2_F_8_: Tb exhibited strong scintillation luminescence centered at 545 nm, and the intensity was about twice than that of CsI (Tl) [18]. The KY_3_F_10_: Pr showed strong scintillation luminescence centered at 260 nm, 490 nm and 610 nm, and the spectral integral intensity was ~2.5 times than that of Bi_4_Ge_3_O_12_ [19]. Although many achievements have been made in these systems, the development of new fluoride scintillators with high performances is still of great significance.

Recently, it was reported that the lead halide perovskites showed strong scintillation intensity and high X-ray imaging quality, which is attributed to the existence of the heavy Pb atom. For example, the indirect X-ray imaging system based on CsPbBr_3_ perovskite has faster response time (200 ns), better X-ray irradiation stability (>40 Gy_air_s^−1^ of X-ray exposure) and higher light output (177,000 photons/MeV) than the traditional GOS: Tb [20]. (CH_3_NH_3_) PbBr_3_ crystals prepared by inverse temperature crystallization exhibited light yield up to 150,000 photons/MeV and sub-nanosecond response time at low temperature [21]. However, these kinds of scintillators exhibit poor environmental stability, which greatly restrict their practical applications. Lanthanide doped fluoride nanoparticles prepared with a low-temperature wet-chemical method possess the advantages of low toxicity, cheap fabrication cost, convenient device processability and adjustable emission wavelengths, which have been studied for high-performance X-ray detection and imaging very recently.

In this work, a series of novel rare-earth (Re = Tb, Eu, Sm, Dy, Ho) doped Pb_4_Lu_3_F_17_ scintillators were developed by a simple hydrothermal method for the first time. The Rietveld refinement results were used to study the host structure. Upon X-ray irradiation, all these scintillators exhibited characteristic 4f-4f transitions. The optimal Tb^3+^ doping concentration was determined to be 30%, which is much higher than other hosts. Moreover, the Ce and Gd ions were verified to be useful for enhancing the scintillation intensity via introducing energy transfer.

## 2. Materials and Methods

**Materials.** The chemical reagents used in the experiments, including Pb(NO_3_)_2_ (99%), Gd(NO_3_)_3_ (99.9%), Lu(NO_3_)_3_ (99.99%), Ce(NO_3_)_3_ (99.95%), Tb(NO_3_)_3_ (99.9%), NH_4_F, ethylene diamine tetraacetic acid (EDTA), and citric acid (CA), were all analytically pure and used directly without further purification. The above reagents, except Pb(NO_3_)_2_, were purchased from Shanghai Aladdin Company. Pb(NO_3_)_2_ was purchased from Tianjin Kemio Chemical Reagent Company (Tianjin, China), anhydrous ethanol was purchased from Hangzhou Shuanglin Chemical Reagent Company (Hangzhou, China), and deionized water was self-produced in the laboratory. 

**Synthesis of Pb_4_Lu_3_F_17_: 30Tb/10Ce/5Gd NCs.** Take the hydrothermal synthesis of Pb_4_Lu_3_F_17_: 30Tb/10Ce/5Gd as an example: first, 8.5 mmol of NH_4_F was dissolved in 15 mL of deionized water and stirred vigorously for 15 min using a magnetic stirrer; then, 2 mmol Pb(NO_3_)_2_, 0.55 × 1.5 mmol Lu(NO_3_)_3_, 0.3 × 1.5 mmol Tb(NO_3_)_3_, 0.1 × 1.5 mmol Ce( NO_3_)_3_, 0.05 × 1.5 mmol Gd(NO_3_)_3_ and 0.18 mmol EDTA were dissolved in 15 mL of deionized water and stirred vigorously for 30 min. Subsequently, the solution with NH_4_F was added to the above mixture and stirred vigorously for another 30 min. Finally, the resulting mixture was transferred to a PTFE liner, set in a stainless steel hydrothermal reactor and placed in an oven. The parameters were set to a heating temperature of 200 °C and a heating time of 12 hours. After the reaction, the product was collected by centrifugation at 1,0000 r/min for 5 min, washed with anhydrous ethanol and centrifuged several times, and finally dried at 60 °C for 3 h. The product powder was obtained after grinding. Other materials were synthesized in a similar way.

**Sample characterizations.** The X-ray diffraction (XRD) patterns of the samples were obtained by a powder diffractometer (Bruker D8 Advance, Saarbrücken, Germany) using Cu-Kα (λ = 1.5405 A) radiation. XRD patterns were recorded in the 2(θ) range 10° to 80° with a scanning rate of 0.02°. The morphology and size of the products were observed by a Scanning electron microscopy (SEM, FEI Tecnai G2 F20, Hillsboro, OR, USA) equipped with an energy dispersive X-ray spectroscope (EDX, Aztec X-Max 80T, Oxford, England). The luminescence and afterglow of the samples were recorded by a X-ray fluorescence spectrometer (Zolix Omni-λ300i, Beijing, China). The X-ray source is a mini MAGPRO X-ray (target material: tungsten, P_max_ = 12 W, V_max_ = 60 kV).

## 3. Results and Discussion

**Figure 1:**
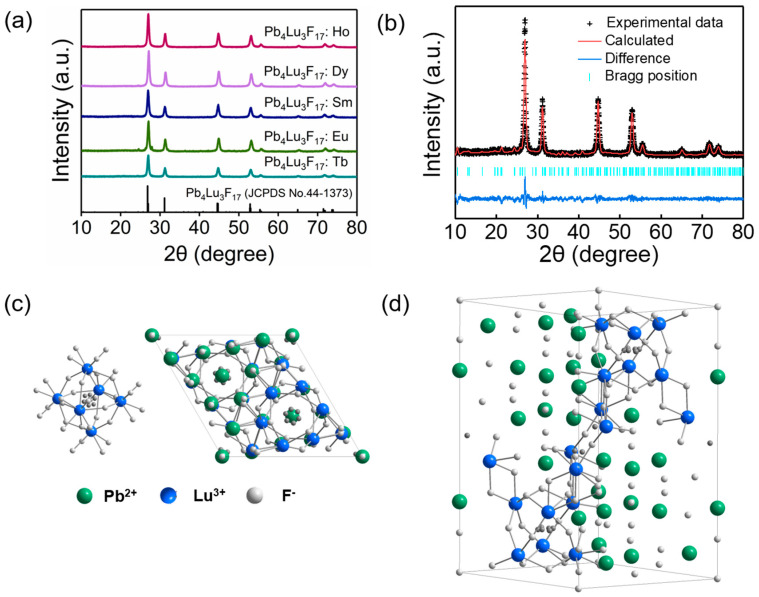
(**a**) XRD patterns of the Pb_4_Lu_3_F_17_: Re (Re = Tb, Eu, Sm, Dy, Ho) samples. (**b**) Experimental (black plus), calculated (red line) and difference (blue line) results of XRD refinement of Pb_4_Lu_3_F_17_: Tb. (**c**) A typical Lu_6_F_37_ cluster, the crystal structure viewed in c-direction. (**d**) Crystal structure of the rhombohedral phase Pb_4_Lu_3_F_17_.

The X-ray diffraction (XRD) patterns of the as-prepared Pb_4_Lu_3_F_17_: Re are shown in Figure 1a. All these products are well indexed with the standard data of pure rhombohedral Pb_4_Lu_3_F_17_ phase (JCPDS No.44-1373). These results suggested that the Re ions were successfully incorporated into the host without the emergence of extra impurity. In order to acquire the crystal structure information of the prepared samples, Rietveld structure refinements of the Pb_4_Lu_3_F_17_: Tb have been performed by the Fullprof program. In the refinements, the crystallographic data of rhombohedral phase Pb_4_Lu_3_F_17_ was used as the initial structural model. The Rietveld refinement results, cell paraments and atomic position coordinates are illustrated in Table 1. The as-obtained goodness of fit parameters were Rwp = 11.7, chi^2^ = 1.84 (Figure 1b), indicating that all atom positions, fraction factors and temperature factors well satisfy the reflection condition [22,23]. Similar Rietveld refinement results were achieved based on the Pb_4_Lu_3_F_17_: Eu product as well (Figure S1 and Table S1).

As shown in Figure 1c, the anions F(1)–F(5) are connected with Lu to form the LuF_8_ (square antiprism) polyhedron. The Pb(1) and Pb(2) atoms are located on the triangular faces of the octahedron. The three-dimensional network of the Pb_4_Lu_3_F_17_ is formed by sharing the external edge of the LuF_8_ polyhedron [24]. The whole ion arrangement in the unit cell is given in Figure 1d. The local symmetry also has a great influence on the luminescence properties, and the reduction of symmetry is beneficial to enhance the luminescence intensity of materials. In the rhombohedral Pb_4_Lu_3_F_17_, the Pb(1) shows C_3_ symmetry, the Pb(2) shows C_1_ symmetry, and the Lu shows C_1_ symmetry [25]. This low local symmetry indicates that the Pb_4_Lu_3_F_17_ is a good potential host for photoluminescence from rare earth ions.

**Figure 2:**
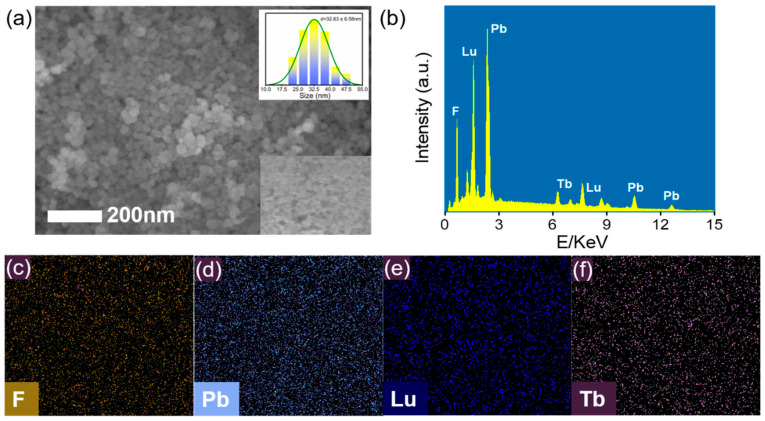
SEM image (**a**), EDX spectrum (**b**), and corresponding EDX mapping results (**c**–**f**) of the EDTA coated Pb_4_Lu_3_F_17_: Tb. Inset in (**a**) is the corresponding histogram of size distributions.

Scanning electron microscopy (SEM) was used to study the morphology of the as-prepared Pb_4_Lu_3_F_17_: Tb using different surfactants. As shown in Figure 2a, the average particle size of the Pb_4_Lu_3_F_17_: Tb nanoparticles was about 32 nm when using EDTA as surfactant. The size was increased to 64 nm when using citric acid (CA) as surfactant (Figure S2). The energy dispersive X-ray (EDX) spectrum revealed the presence of Pb, Lu, F and Tb elements in the final product (Figure 2b), and the EDX mapping results suggested the uniform distribution of these elements in the particles. As shown in Figure S3, the scintillation intensity of the EDTA coated nanoparticles with smaller size was much stronger than that of CA coated nanoparticles, which was probably attributed to the EDTA coated nanoparticles having higher crystallinity than the CA coated nanoparticles [26].

**Figure 3:**
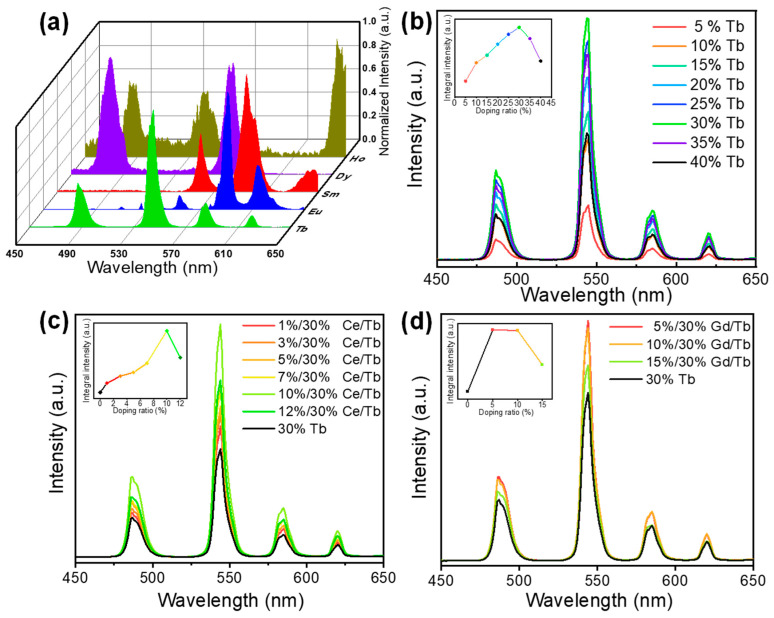
(**a**) Normalization scintillation spectra of the Pb_4_Lu_3_F_17_: Re^3+^ (Re = Tb, Eu, Sm, Dy, Ho). (**b**) Scintillation spectra of Pb_4_Lu_3_F_17_: Tb with different Tb^3+^ doping concentrations (x = 5, 10, 15, 20, 25, 30, 35, 40 mol%). (**c**) Scintillation spectra of the Pb_4_Lu_3_F_17_: 30Tb/yCe (y = 1, 3, 5, 7, 10, 12 mol%). (**d**) Scintillation spectra of the Pb_4_Lu_3_F_17_: 30Tb/yGd (y = 5, 10, 15 mol%).

The normalized X-ray luminescence spectra of the Pb_4_Lu_3_F_17_: Re (Re = Tb, Eu, Sm, Dy, Ho) are presented in Figure 3a. These scintillating nanoparticles exhibited characteristic emission peaks corresponding to different energy level transitions of Re^3+^ ions. Taking the Pb_4_Lu_3_F_17_: Tb as an example: under the X-ray irradiation at 50 KV, the sample showed typical emissions of Tb^3+^ centered at 487 nm, 545 nm, 587 nm and 620 nm corresponding to the ^5^D_4_→^7^F_j_ (j = 3–6) transitions. The green emission at 545 nm (^5^D_4_-^7^F_5_) is a magnetic dipole transition with ΔJ = ±1, which is more intense than the other transitions [27]. As shown in Figure 3b, the luminescence intensity of the Pb_4_Lu_3_F_17_: Tb was increased when the doping concentration was changed from 5% to 30%, and then significantly decreased with a further increase in the doping concentration to 40%. This can be attributed to the typical concentration quenching effect [28]. Similarly, upon X-ray irradiation, the typical emissions of Sm, Eu, Dy, Ho were recorded as well (Figure 3a). As shown in Figure 3c,d, the luminescence intensity of Tb could be further improved by incorporating Ce or Gd. The optimal doping concentrations of Ce and Gd were measured to be about 10% and 5%, respectively. It should be noted that the X-ray luminescence intensity was decreased when simultaneously cooping 30 Tb/10 Ce/5 Gd in the Pb_4_Lu_3_F_17_ host (Figure S4), which might be attributed to the generation of TbF_3_ impurity phase (Figure S5) followed by the reduced Tb^3+^ concentration in the Pb_4_Lu_3_F_17_ host.

**Figure 4:**
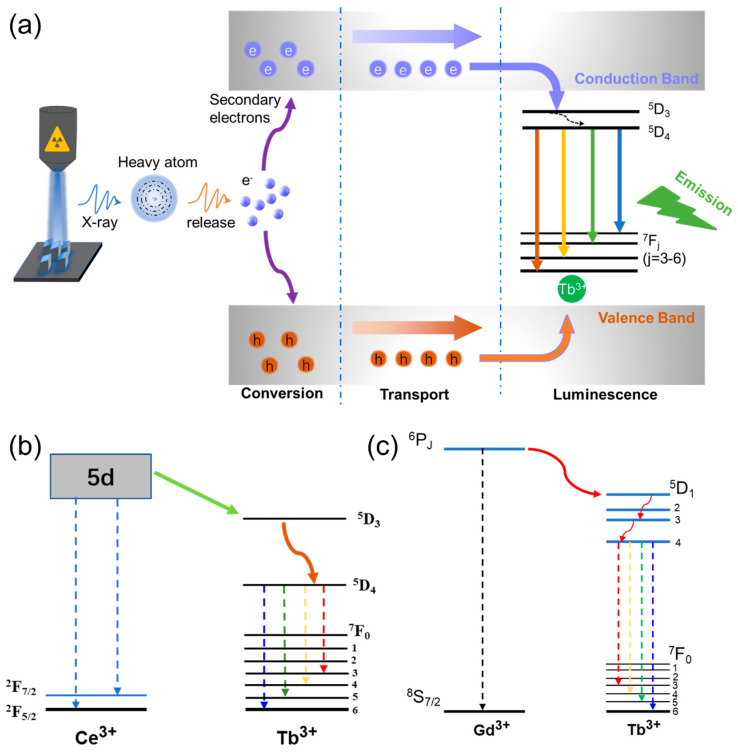
(**a**) Luminescence mechanism of the Pb_4_Lu_3_F_17_: Tb NCs upon X-ray irradiation. (**b**) Energy level transition diagram of Ce^3+^ and Tb^3+^. (**c**) Energy level transition diagram of Gd^3+^ and Tb^3+^.

The proposed luminous mechanism is shown in Figure 4a. The interaction between X-ray photons and heavy atoms of Lu and Pb leads to the generation of hot electrons through the photoelectric effect. Then, massive secondary electrons are generated via electron–electron scattering and the Auger process. Finally, these low-energy electrons are transported through the conduction band to the luminescence center of the Tb^3+^ ion. Figure 4b,c show the proposed energy transfer mechanism for the above enhanced luminescence intensity. The Ce: 5d and Gd: ^6^P_j_ states could enhance the electrons’ population efficiency in the Tb: ^5^D_3_ level, which leads to the improved X-ray luminescence intensity [29,30].

**Figure 5:**
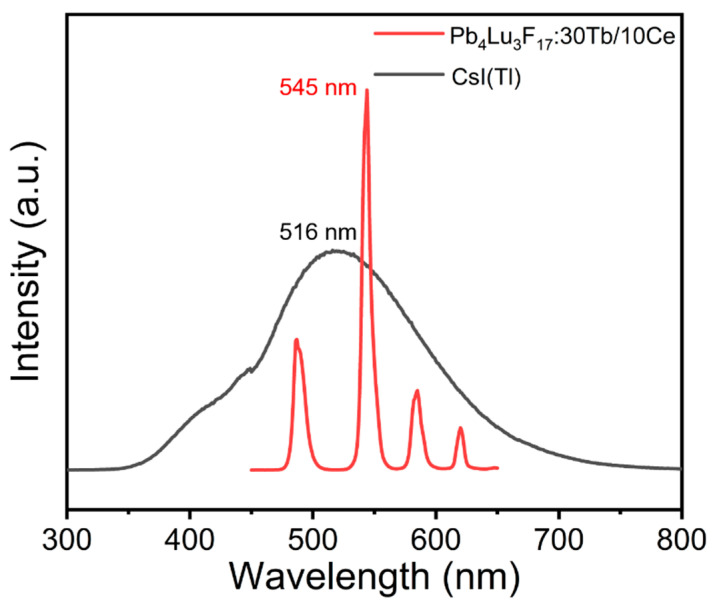
X-ray excited emission spectra of the Pb_4_Lu_3_F_17_: 30Tb10Ce and CsI (Tl).

Compared with the conventional commercial inorganic scintillator CsI (TI), the Pb_4_Lu_3_F_17_: Tb/Ce nanoparticles have a main emission peak of 545 nm, which is close to the conventional commercial inorganic scintillation of CsI (TI) (516 nm) and can be well matched with the silicon photodiode’s sensitive wavelength band (520 nm–580 nm). As shown in Figure 5, the integrated scintillation intensity of the Pb_4_Lu_3_F_17_: Tb/Ce is about 16.8% of the commercial CsI (Tl) single crystal. Through designing crystal structure, such as core/shell, the scintillating intensity of this new kind of scintillator might be further improved, which will be used for X-ray detection and imaging.

## 4. Conclusions

In conclusion: a series of the Pb_4_Lu_3_F_17_: Re (Re = Tb, Eu, Sm, Dy, Ho) were prepared by a simple hydrothermal method. Our results revealed that the EDTA is a better surfactant than CA for scintillation intensity of the Pb_4_Lu_3_F_17_: Re. All the doped rare earth ions in the Pb_4_Lu_3_F_17_ host show their corresponding characteristic emissions. The optimal Tb^3+^ doping concentration is verified to be 30 mol%, which is much higher than most hosts. The integrated scintillation intensity of the Pb_4_Lu_3_F_17_: Tb/Ce is about 16.8% of the commercial CsI (Tl) single crystal.

## Figures and Tables

**Table 1:** The Rietveld refinement results, cell paraments and atomic position coordinates for the Pb_4_Lu_3_F_17_: Tb.

Formula			Pb_4_Lu_3_F_17_: Tb			
Crystal system			rhombohedral			
Density (g/cm^3^)			7.144			
Space-group			R3 (148)			
a (Å) = b (Å)			10.72442			
c (Å)			19.86123			
α = β (°)			90			
γ (°)			120			
Rwp (%)			11.7			
chi^2^			1.84			
Atoms	X	Y	Z	B	Occ.	Site
Pb (1)	0	0	0.2586	1.658		6
Pb (2)	0.2292	0.0369	0.0836	2.163		18
Lu	0.09	0.6127	0.0835	0.774		18
F (1)	0.036	0.767	0.0376	1.5		18
F (2)	0.426	0.291	0.1101	1.5		18
F (3)	0.475	0.082	0.321	1.5		18
F (4)	0.203	0.485	0.341	1.5		18
F (5)	0.267	0.392	0.1735	1..5		18
F (6)	0	0	0.145	1.5		6
F (7)	0	0	0	1.5		3
F (8)	0.02	0.057	0.502	1.5	0.167	18

## Data Availability

Not applicable.

## Supplementary Materials

The following supporting information can be downloaded at: https://www.mdpi.com/article/10.3390/ma16031147/s1.
