# Supplementary material for: Scintillation Properties of Lanthanide Doped Pb4Lu3F17 Nanoparticles
Source: Materials (Basel). 2023 Jan 29;16(3):1147. doi: 10.3390/ma16031147 (PMC9919652; doi:10.3390/ma16031147)
Supplement: Supplementary file 1 [file materials-16-01147-s001.zip › materials-2103945-supplementary.pdf]

materials MDPI

Supporting Information

Scintillation Properties of Lanthanide Doped $Pb_4Lu_3F_{17}$ Nanoparticles

Peng Qiao [1,2], Yiheng Ping [3], Hongping Ma [3,*] and Lei Lei [4,*]

1 Zhejiang Academy of Special Equipment Science, Hangzhou 310018, China
2 Key Laboratory of Special Equipment Safety Testing Technology of Zhejiang Province, Hangzhou 310018, China
3 School of Mechanical and Energy Engineering, Zhejiang University of Science and Technology, Hangzhou 310023, China
4 College of Optical and Electronic Technology, China Jiliang University, Hangzhou 310018, China
* Correspondence: hongpingma@163.com (H.M.); leilei@cjlu.edu.cn (L.L.)

Table S1. The Rietveld refinement results, cell paraments and atomic position coordinates for the $Pb_4Lu_3F_{17}$: Eu.

Formula	$Pb_4Lu_3F_{17}$: Eu
Crystal system	rhombohedral
Density (g/cm^3)	7.144
Space-group	R3 (148)
a (Å) = b (Å)	10.72943
c (Å)	19.84274
$\alpha = \beta$ (°)	90
γ (°)	120
Rwp (%)	11.7
chi^2	2.84

Atoms	X	Y	Z	B	Occ.	Site
Pb (1)	0	0	0.2586	1.658		6
Pb (2)	0.2292	0.0369	0.0836	2.163		18
Lu	0.09	0.6127	0.0835	0.774		18
F (1)	0.036	0.767	0.0376	1.5		18
F (2)	0.426	0.291	0.1101	1.5		18
F (3)	0.475	0.082	0.321	1.5		18
F (4)	0.203	0.485	0.341	1.5		18
F (5)	0.267	0.392	0.1735	1..5		18
F (6)	0	0	0.145	1.5		6
F (7)	0	0	0	1.5		3
F (8)	0.02	0.057	0.502	1.5	0.167	18

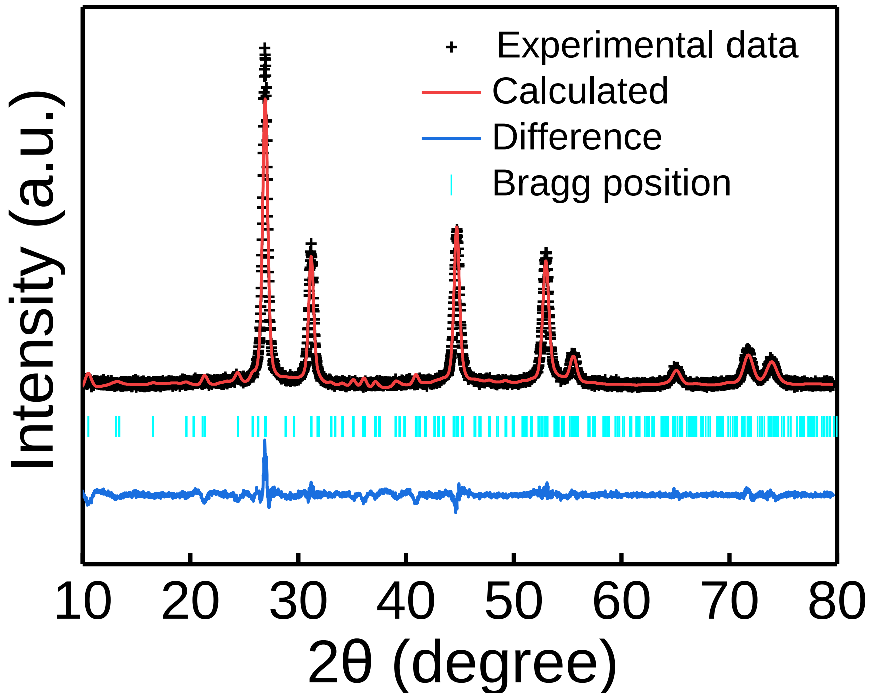

Figure S1. Experimental (black plus), calculated (red line) and difference (blue line) results of XRD refinement of $Pb_4Lu_3F_{17}$: Eu.

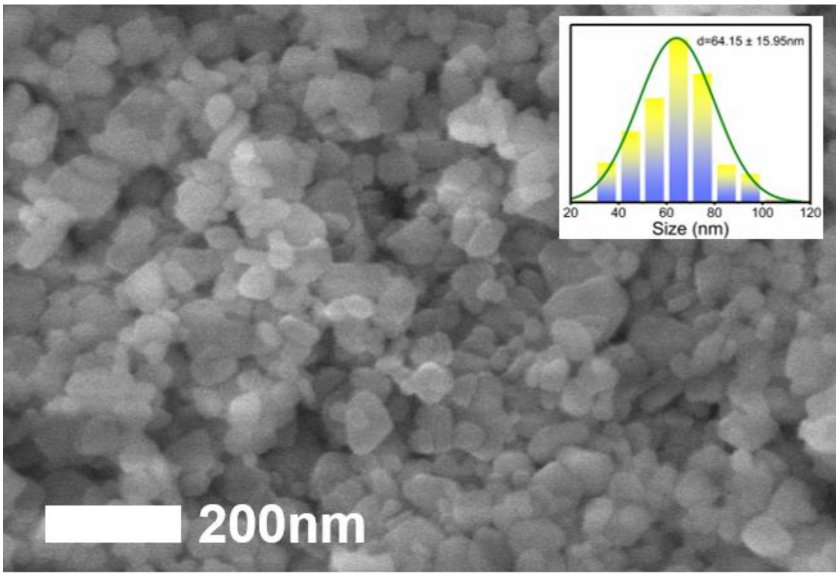

Figure S2. SEM image and size distributions of the $Pb_4Lu_3F_{17}$: Tb using CA as surfactant.

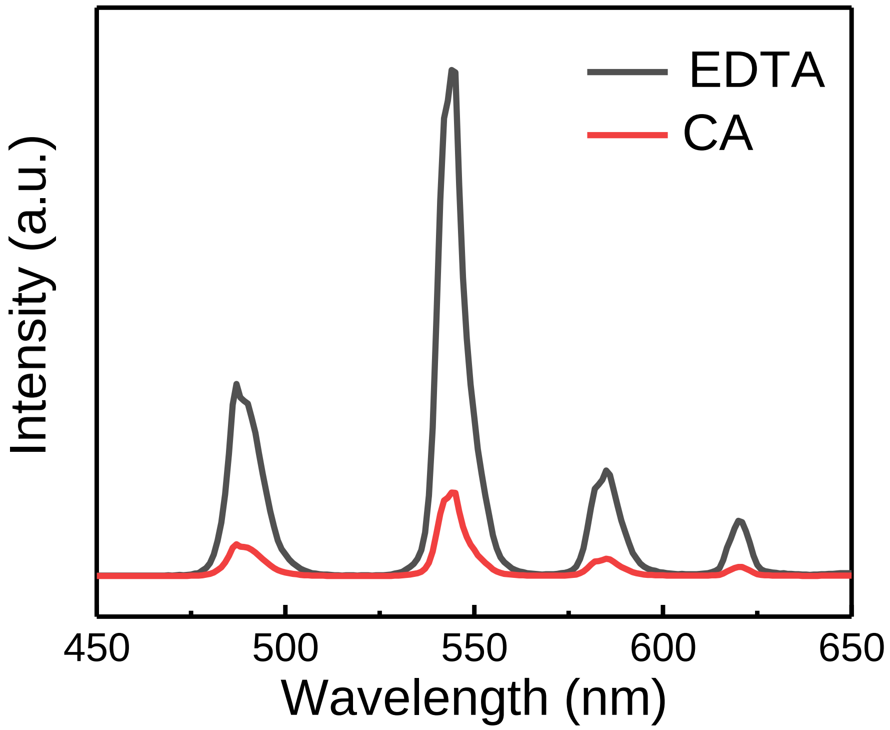

Figure S3. Scintillation spectra of the $Pb_4Lu_3F_{17}$: Tb using EDTA and CA, respectively.

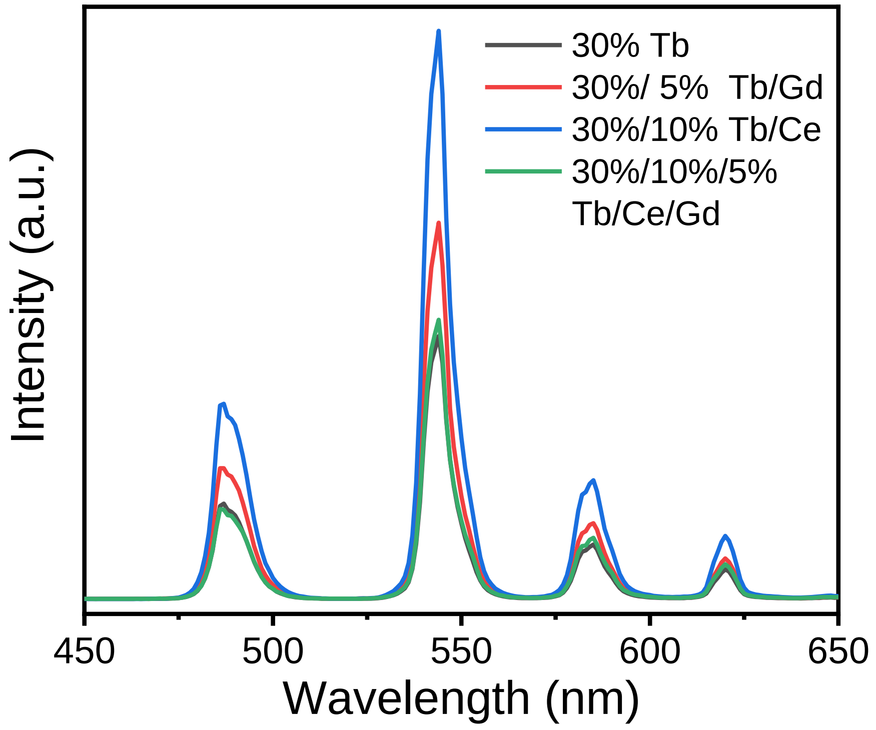

Figure S4. Emission spectrum of the $Pb_4Lu_3F_{17}$: 30Tb10Ce5Gd.

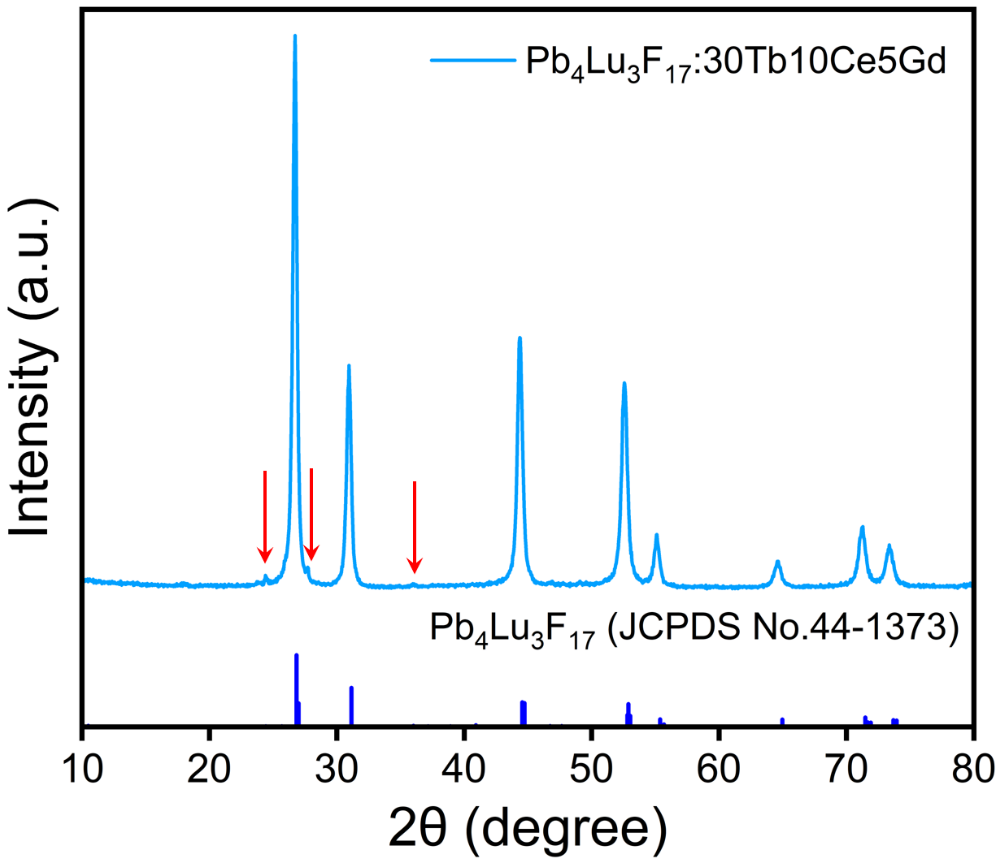

Figure S5. XRD patterns of the $Pb_4Lu_3F_{17}$: 30Tb10Ce5Gd.
